# Serum Albumin Is Associated With Higher Inflammation and Carotid Atherosclerosis in Treated Human Immunodeficiency Virus Infection

**DOI:** 10.1093/ofid/ofy291

**Published:** 2018-11-17

**Authors:** Sahera Dirajlal-Fargo, Manjusha Kulkarni, Emily Bowman, Lingpeng Shan, Abdus Sattar, Nicholas Funderburg, Grace A McComsey

**Affiliations:** 1 University Hospitals Cleveland Medical Center, Ohio; 2 Rainbow Babies and Children’s Hospital, Cleveland, Ohio; 3 Case Western Reserve University, Cleveland, Ohio; 4 Ohio State University School of Health and Rehabilitation Sciences, Columbus

**Keywords:** albumin, HIV, cardiovascular disease, immune activation, gut integrity

## Abstract

**Background:**

This study was conducted to explore the associations between serum albumin and markers of inflammation and cardiovascular disease in treated human immunodeficiency virus (HIV)-infected adults.

**Methods:**

We conducted a nested study within in the SATURN-HIV trial in which 147 HIV^+^ adults on stable antiretroviral therapy were (1) virally suppressed, (2) had a low-density lipoprotein (LDL)-cholesterol level <130 mg/dL, and (3) were randomized to 10 mg daily rosuvastatin or placebo. Measures of serum albumin, carotid intima media thickness ([cIMT] surrogate marker of atherosclerosis), inflammation, T cells, monocyte activation, and gut integrity were assessed at baseline, 48 and 96 weeks later. Spearman correlations and linear mixed-effect models were used to assess associations with serum albumin.

**Results:**

Mean age was 45 years, 80% of participants were male, and 69% were African American. Mean serum albumin was similar between the groups at all time points (4.01–4.09 g/dL in statin arm vs 4.02–4.11 g/dL in placebo arm; *P* = .08–0.35). Lower baseline serum albumin significantly predicted larger changes in cIMT, interleukin 6, D-dimer, tumor necrosis factor α receptor 1, fibrinogen, and high-sensitivity C-reactive protein (*P* ≤ .03) over 96 weeks independently of statin therapy. After adjusting for age, gender, smoking, body mass index, creatinine clearance, and LDL cholesterol, every 1 g/dL decrease in serum albumin at baseline remained associated with a 0.05-mm increase in cIMT over 96 weeks (*P* = .05).

**Conclusions:**

Lower serum albumin in controlled HIV is associated with higher markers of chronic inflammation and hypercoagulation, which could explain the prior observation that serum albumin predicts nonacquired immune deficiency syndrome events in HIV. Serum albumin may predict progression of carotid atherosclerosis independent of traditional risk factors.

Despite control of human immunodeficiency virus (HIV)-1 replication by antiretroviral therapy (ART), HIV^+^ adults remain at a higher risk of comorbidities including cardiovascular disease (CVD). Impaired metabolic pathways and persistent inflammation and immune activation are potential contributing factors to these increased comorbidities. Identification of novel biomarkers and unrecognized risk factors that predict the risk of or that could be modified to improve the outcome of CVD in asymptomatic HIV-infected patients is imperative.

In HIV-uninfected individuals, serum albumin has been recognized as an independent prognostic factor in many CVDs. Evidence suggests that the risks associated with ischemic heart disease [1], heart failure [2, 3], atrial fibrillation [4], stroke [5], and venous thromboembolism [6] are inversely related to serum albumin levels in uninfected individuals, independent of traditional risk factors, body mass index (BMI), and inflammation. Serum albumin may play a role in prevention of CVD through its antioxidant, anti-inflammatory, and antiplatelet aggregation activities [7–13].

In HIV infection, low serum albumin has been associated with disease progression and high-risk mortality in patients initiating ART [14–16]. Findings from the Strategic Timing of Antiretroviral Treatment (START) study demonstrated that in healthy adults with early stage HIV infection, serum albumin predicted future non-acquired immune deficiency syndrome (AIDS)-related events [17]. These findings were recently confirmed in the large D:A:D cohort; serum albumin was associated with the development of CVD and non-AIDS malignancies over time in over 16000 individuals [18]. These findings raise important questions related to why serum albumin levels predict clinical events in HIV and whether adjusting for inflammation markers would abolish this association.

Our objectives in this study were to examine the associations between serum albumin and markers of inflammation, immune activation, coagulation, and gut integrity to explore the pathways by which serum albumin may modulate CVD, using carotid intima media thickness (cIMT) as a surrogate marker of CVD. This is a secondary analysis of SATURN-HIV, a 96-week statin study in HIV-infected participants on stable ART.

## METHODS

### Study Design

SATURN-HIV is a randomized, double-blind, placebo-controlled study designed to measure the effect of rosuvastatin on markers of cardiovascular risk, skeletal health, and immune activation in HIV-infected adults. The results presented herein represent a secondary analysis that assessed serum albumin changes and its associations over 96 weeks. The study is registered on clinicaltrials.gov (NCT01218802). The study was approved by the Institutional Review Board of University Hospitals Case Medical Center (Cleveland, OH), and all subjects signed a written consent before enrollment. The study protocol conforms to the ethical guidelines of 1975 Declaration of Helsinki. Randomization was conducted as 1:1 to active rosuvastatin 10 mg daily versus matching placebo, and it was stratified by protease inhibitor (PI) use. Study drugs (active and placebo) were provided by AstraZeneca.

### Study Population

All participants were ≥18 years of age with HIV-1 infection, on stable ART for at least 3 months, with cumulative ART duration of at least 6 months, HIV-1 ribonucleic acid (RNA) <1000 copies/mL, and fasting low-density lipoprotein cholesterol (LDL-C) ≤130 mg/dL and triglyceride ≤500 mg/dL. Additional entry criteria included evidence of either heightened T-cell activation, identified as the proportion of CD8^+^ T cells that expressed CD38^+^HLA-DR^+^ ≥19%, or levels of high-sensitivity C-reactive protein (hsCRP) ≥2 mg/L. Participants were excluded if they had a history of coronary disease or diabetes, were pregnant or lactating, or had an active infectious or inflammatory condition.

### Study Evaluation

Self-reported demographics and medical history were obtained along with a targeted physical exam including height, weight, waist, and hip measurements. At entry and weeks 24, 48, and 96, fasting (>12 hours) blood draws were obtained for real-time measurements of renal and lipid profiles, glucose, and insulin levels. In addition, blood was processed, and plasma, serum, and peripheral blood mononuclear cells were stored for measurements of immune markers and arginine metabolites. Human immunodeficiency virus-1 RNA levels and CD4^+^ cell counts were drawn as part of clinical care, and measures closest to study visits were used for analysis.

#### Serum Albumin

Serum albumin was measured on fresh serum within 8 hours of collection, with a Beckman AU Bromcresol green (BCG) dye-binding assay. At pH 4.2, bromocresol green reacted with albumin to form an intense green complex. The absorbance of the albumin-BCG complex was measured bichromatically (600/800 nm) and was proportional to the albumin concentration in the sample

#### Inflammation and Soluble Immune Activation Markers

Several biomarkers of systemic inflammation and immune activation were measured in plasma, including interleukin-6 (IL-6), soluble tumor necrosis factor-α receptors I and II (sTNFR-I and II), and interferon γ-inducible protein-10 (IP-10), soluble vascular cell adhesion molecule-1 (sVCAM-1), soluble intercellular adhesion molecule-1 (sICAM-1), and 2 soluble markers of monocyte activation (sCD14 and sCD163). All were determined by enzyme-linked immunosorbent assay ([ELISA] R&D Systems, Minneapolis, MN). High-sensitivity CRP and fibrinogen were measured by particle-enhanced immunonephelometric assay on a BNII nephelometer (Siemens, Munich, Germany). D-dimer was determined by immunoturbidometric assay on a STA-R Coagulation Analyzer (DiagnosticaStago).

#### Monocytes and T-Cell Measurements

Monocytes and T-cells were phenotyped by flow cytometry as previously described [19]. Monocyte subsets (CD14^+^CD16^+^, CD14^dim^CD16^+^, and CD14^+^CD16^−^) were each quantified as a percentage of the overall monocyte population. T-cell activation was quantified as the percentage of CD4^+^ or CD8^+^ cells that expressed both CD38 and HLA-DR. Entry CD8^+^ activation was measured from frozen peripheral blood mononuclear cells.

#### Gut Integrity Markers

Levels of zonulin-1 (ALPCO, Salem, NH), intestinal fatty acid binding protein (I-FABP) (R&D Systems human FABP2 DuoSet ELISA system, Minneapolis, MN), and liposaccharide binding protein (LBP) (Hycult Biotech, Plymouth Meeting, PA) were measured by ELISAs.

#### Cardiovascular Measures

Mean-mean common carotid artery intima media thickness was measured at baseline and at weeks 48 and 96 (as described previously [20]) offline by a single reader using semi-automated edge-detection software (Medical Imaging Applications, Coralville, IA).

### Statistical Analysis

This is an exploratory analysis to assist in developing hypotheses for future confirmatory studies. Demographics, clinical characteristics, fasting metabolic parameters, and inflammatory and coagulation markers were described by study groups. Continuous biomarkers or variables were reported using the mean and standard deviation, whereas categorical variables were reported with frequency and percentage. Descriptive statistics, *t* tests, and χ^2^ tests were used for comparing baseline HIV parameters, cardiovascular risk factors, and inflammation and immune activation gut integrity markers between the 2 groups.

Spearman correlation analyses were used to assess the relationships between serum albumin and markers of inflammation, immune activation, coagulation, gut integrity, and cardiovascular risk. Linear mixed-effects models were used in studying associations between serum albumin over 96 weeks and inflammatory markers, monocyte activation, HIV-related factors, and CVD risk factors. A linear mixed-effects model with random intercept and slope was also used in studying association between changes in cIMT (over 96 weeks, response variable) and serum albumin after controlling for traditional CVD risk factors, renal function, and inflammatory markers. We specified unstructured covariance matrix for random effects of the linear mixed-effects models. We assumed that missing outcome values in the linear mixed-effects model were missing at random. The models were initially performed including all participants and all available data. The results of the analyses including all participants did not differ from the sensitivity analyses performed including only participants with undetectable viral load at baseline; therefore, only the former data are presented. All of the statistical analyses were performed using SAS 9.4 and R 3.4.0.

## RESULTS

### Baseline Characteristics

All of the 147 participants from the SATURN-HIV study were included in this analysis, 119 of whom completed all measures. Demographic information and baseline characteristics are shown in Table 1 and were similar between groups. Mean age was 45 years (standard deviation = 9), 79% were male, and 68% were African American. Mean BMI was 28 kg/m^2^ (standard deviation = 6), lipid profiles were low risk, and 63% were current smokers. Mean CD4 T-cell count was 640 (300) cells/µL. All participants were on ART by design; 50% received a PI, 88% received tenofovir, and 77% had undetectable viral load (<50 copies/mL). Seven subjects had active hepatitis B (statin, n = 3 vs placebo n = 4), and 12 subjects had active hepatitis C (statin n = 5 vs placebo n = 7). All inflammation markers, immune activation markers, and gut integrity or microbial translocation markers (I-FABP, zonulin, and LBP) were similar between the study arms.

**Table 1.** Baseline Characteristics of Enrolled HIV^+^ Patients

Characteristics	Overall	Rosuvastatin	Placebo	*P* Value
Sample size	147	72	75	
Demographics				
Females (%)	32 (21.8)	14 (19.4)	18 (24.0)	.64
African American (%)	100 (68.0)	50 (69.4)	50 (66.7)	.99
Current smoking (%)	93 (63.3)	43 (59.7)	50 (66.7)	.40
Age^a^	45.41 (9.93)	45.39 (9.14)	45.42 (10.70)	.98
Metabolic and Cardiovascular Parameters				
Body mass index (kg/m^2^)	28.07 (6.49)	28.05 (6.35)	28.09 (6.66)	.97
Systolic blood pressure (mmHg)	122.66 (16.44)	124.56 (17.91)	120.84 (14.79)	.17
Diastolic blood pressure (mmHg)	78.66 (9.67)	79.53 (9.33)	77.83 (9.98)	.29
Framingham score	4.95 (4.56)	5.03 (4.80)	4.88 (4.34)	.85
Creatinine clearance (mL/min)	119.37 (43.19)	119.71 (42.63)	119.05 (44.00)	.93
Total cholesterol (mg/dL)	172.24 (28.90)	166.90 (29.32)	177.36 (27.73)	.03
LDL cholesterol (mg/dL)	94.35 (24.93)	92.15 (24.17)	96.47 (25.63)	.30
HDL cholesterol (mg/dL)	48.63 (15.86)	48.56 (16.44)	48.71 (15.40)	.95
Serum albumin (g/dL)	4.05 (0.34)	4.01 (0.36)	4.09 (0.33)	.17
Hepatitis				
Active hepatitis B (%)	7 (5%)	3 (4%)	4 (5%)	>.99
Active hepatitis C (%)	12 (8%)	5 (7%)	7 (9%)	>.99
Inflammation and Immune Activation				
Interleukin-6 (pg/mL)	4.48 (9.36)	5.42 (13.14)	3.57 (2.33)	.23
hsCRP^c^ (μg/mL)	2.20 [0.90, 4.50]	2.30 [0.90, 5.00]	2.10 [0.90, 4.25]	.49
ICAM (ng/mL)	245.52 (109.62)	248.10 (115.68)	243.04 (104.19)	.78
VCAM (ng/mL)	722.40 (295.58)	760.64 (322.79)	685.70 (263.85)	.13
D-dimer (μg/mL)	0.30 (0.36)	0.32 (0.39)	0.27 (0.32)	.42
Fibrinogen (mg/dL)	423.11 (129.50)	433.56 (145.36)	413.08 (112.28)	.34
TNFα-receptor I (pg/mL)	1845.75 (812.91)	1876.32 (820.29)	1816.40 (810.19)	.66
TNFα-receptor II (pg/mL)	2337.56 (836.68)	2461.42 (878.05)	2218.65 (782.42)	.08
IP-10 (pg/mL)	219.40 [155.61, 347.12]	224.63 [170.21, 351.76]	219.40 [144.19, 336.55]	.48
sCD14 (ng/mL)	2267.40 (1361.37)	2440.45 (1833.86)	2101.28 (610.56)	.13
sCD163 (ng/mL)	734.49 (361.31)	747.51 (392.81)	721.99 (330.44)	.67
CD14^+^CD16^+^ monocytes (%)	12.19 (6.07)	12.95 (6.11)	11.48 (5.99)	.15
CD4^+^CD38^+^HLA-DR^+^ T cells (%)	5.68 (2.93)	5.97 (3.34)	5.41 (2.47)	.25
CD8^+^CD38^+^HLA-DR^+^ T cells (%)	14.83 (9.41)	16.15 (10.26)	13.60 (8.43)	.11
Gut Integrity and Microbial Translocation				
Zonulin (ng/mL)	9250.35 (7261.71)	10 259.15 (9127.96)	8206.76 (4451.71)	.13
Intestinal fatty acid binding protein (pg/mL)	4529.99 (3206.82)	4561.53 (3186.75)	4497.36 (3254.97)	.91
Lipopolysaccharide binding protein (µg/mL)	20.80 (10.85)	21.11 (10.91)	20.49 (10.87)	.76
Surrogate Markers of CVD				
Carotid intima media thickness (mm)	0.70 (0.14)	0.72 (0.16)	0.69 (0.11)	.18
HIV Variables				
CD4 (cells/µL)	639.88 (300)	636 (314)	644 (287)	.88
HIV <50 copies/mL (%)	113 (77%)	33 (48%)	56 (78%)	.79
ART duration (months)	86 (63)	87 (62)	84 (64)	.78
TDF use (%)	130 (88%)	66 (88%)	64 (89%)	>.99
Protease inhibitors (%)	73 (50%)	36 (50%)	37 (49%)	.79

Abbreviations: ART, antiretroviral therapy; CVD, cardiovascular disease; HDL, high-density lipoprotein; HIV, human immunodeficiency virus; hsCRP, high-sensitivity C-reactive protein; ICAM, intercellular adhesion molecule; IP-10, interferon γ-inducible protein; LDL, low-density lipoprotein; sCD14, soluble CD14; TDF, tenofovir disoproxil fumarate; TNF, tumor necrosis factor; VCAM, vascular cell adhesion molecule.

^a^All normally distributed continuous variables are summarized as mean (standard deviation).

^b^Categorical variables are summarized with counts, percentages.

^c^All skewed continuous variables are summarized as median [1st quartile, 3rd quartile].

### Changes in Serum Albumin

**Figure 1.**
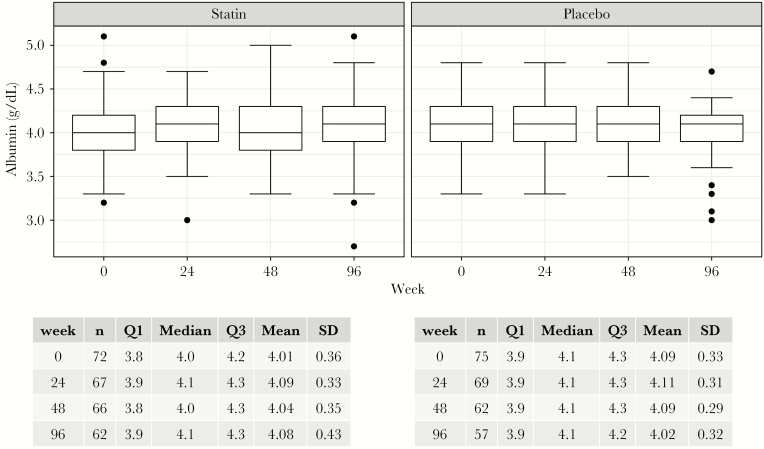
Serum albumin concentration over the study period.

As seen in Figure 1, mean serum albumin was similar between the statin and placebo arm at all time points (4.05–4.08 g/dL in statin arm vs 4.01–4.11 g/dL in placebo arm; *P* = .08–.35). At baseline, only 4 participants (3%) had hypoalbuminemia (serum baseline <3.5 g/dL).

### Carotid Intima Media Thickness and Baseline Serum Albumin

**Figure 2.**
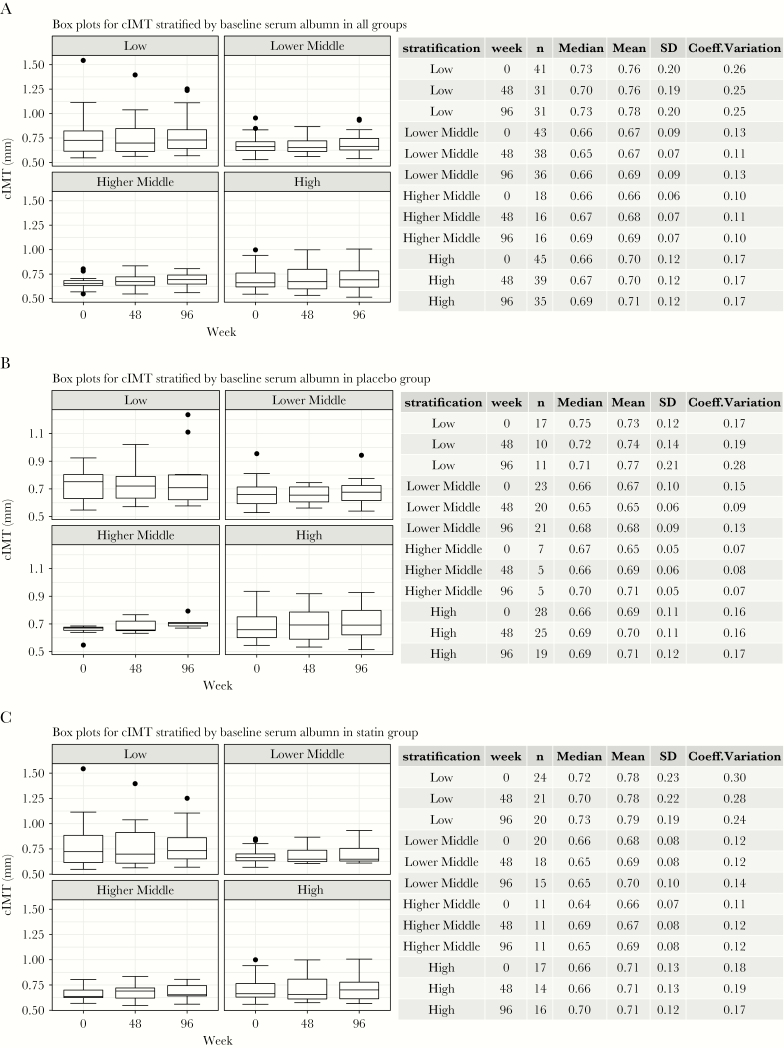
Carotid intima media thickness (cIMT) stratified by serum albumin at each study time point and by randomization group. (a) Box plots for cIMT stratified by baseline serum albumin levels in all groups. (b) Box plots for cIMT stratified by baseline serum albumin levels in the placebo group. (c) Box plots for cIMT stratified by baseline serum albumin levels in the statin group. Box plots representing median values with whiskers representing the upper and lower quartiles.

Changes in cIMT over the study period were stratified by baseline serum albumin at all time points of the study. Baseline serum albumin was categorized by quartiles (<3.8 g/dL [low]; 3.8 g/dL–4.1 g/dL [lower middle]; 4.1 g/d–4.3 g/dL [higher middle]; and >4.3 g/dL [high]). As shown in Figure 2, at each time points and regardless of the randomization arm, the highest cIMT measures were found at the lowest serum albumin values.

### Baseline Serum Albumin as a Predictor of Change in Inflammatory and Cardiovascular Markers Over 96 Weeks

In linear mixed-effects model, baseline lower serum albumin was independently associated with higher levels of IL-6, hsCRP, D-dimer, fibrinogen, TNFα receptor I, and larger changes in cIMT at 96 weeks even after adjusting for statin over the study period (Table 2).

**Table 2.** Analysis of Baseline Serum Albumin as Predictor of Change in Inflammatory, Immune Activation, and Gut Integrity Makers Over 96 Weeks

Outcome	Parameter	β^ (SE)	95% CI of β^	*P* Value
Systemic Inflammation Makersa				
Interleukin-6 (pg/mL)	Albumin	−2.16 (0.94)	(−4.01 to −0.31)	**.03**
	Statin	−0.02 (0.02)	(−0.05 to 0.02)	.36
hsCRP (μg/mL)	Albumin	−34.59 (2.74)	(−39.98 to −29.21)	**<.001**
	Statin^b^	<0.001	-	-
ICAM (ng/mL)	Albumin	19.18 (25.66)	(−31.35 to 69.72)	.46
	Statin	−0.14 (0.12)	(−0.37 to 0.10)	.26
VCAM (ng/mL)	Albumin	110.05 (68.04)	(−23.96 to 244.06)	.11
	Statin	−1.07 (0.44)	(−1.93 to −0.21)	**.02**
D-dimer (μg/mL)	Albumin	−0.29 (0.08)	(−0.44 to −0.13)	**<.001**
	Statin	0.002 (0.002)	(−0.002 to 0.006)	.28
Fibrinogen (mg/dL)	Albumin	−113.75 (18.88)	(−150.94 to −76.56)	**<.001**
	Statin	−0.20 (0.19)	(−0.58 to 0.17)	.29
TNFα receptor I (pg/mL)	Albumin	−275.42 (124.62)	(−520.87 to −29.96)	**.02**
	Statin	0.35 (1.12)	(−1.86 to 2.56)	.75
TNFα receptor II (pg/mL)	Albumin	−158.98 (178.61)	(−510.78 to 192.81)	.37
	Statin	−1.92 (1.52)	(−4.91 to 1.07)	.21
IP-10 (pg/mL)	Albumin	28.73 (50.06)	(−69.87 to 127.33)	.57
	Statin	0.32 (0.46)	(−1.23 to 0.59)	.49
Immune and Monocyte Activation Markers				
sCD14 (ng/mL)	Albumin	−210.51 (143.52)	(−493.20 to 72.18)	.14
	Statin	−2.71 (2.70)	(−8.04 to 2.62)	.32
sCD163 (ng/mL)	Albumin	20.28 (92.76)	(−162.43 to 202.99)	.83
	Statin	−0.11 (0.83)	(−1.74 to 1.51)	.89
CD14^+^CD16^+^ monocytes (%)	Albumin	−1.18 (1.08)	(−3.30 to 0.94)	.27
	Statin	−0.020 (0.010)	(−0.0404 to 0.0008)	.06
T-Cell Activation Markers				
CD4^+^CD38^+^HLA-DR^+^ T cells (%)	Albumin	0.62 (0.67)	(−0.71 to 1.95)	.31
	Statin	−0.024 (0.009)	(−0.041 to 0.007)	**<.01**
CD8^+^CD38^+^HLA-DR^+^ T cells (%)	Albumin	3.58 (1.93)	(−0.22 to 7.38)	.06
	Statin	−0.08 (0.03)	(−0.13 to −0.02)	**<.01**
Microbial Translocation and Gut Integrity Markers				
Zonulin (ng/mL)	Albumin	1045.77 (1961.36)	(−2838.95 to 4930.49)	.59
	Statin	−3.36 (40.86)	(−83.86 to 77.14)	.09
Intestinal Fatty Acid Binding Protein (pg/mL)	Albumin	−727.96 (732.65)	(−2179.07 to 723.15)	.32
	Statin	−13.46 (10.67)	(−34.60 to 7.68)	.21
Lipopolysaccharide binding protein (µg/mL)	Albumin	0.80 (2.15)	(−3.47 to 5.06)	.71
	Statin	0.02 (0.04)	(−0.05 to 0.09)	.64
Cardiovascular Disease Markers				
Framingham score	Albumin	0.31 (1.04)	(−1.73 to 2.36)	.76
	Statin	−0.004 (0.006)	(−0.016 to 0.007)	.45
Carotid intima media thickness (mm)	Albumin	−0.07 (0.03)	(−0.130 to −0.007)	**.03**
	Statin	−0.0002 (0.00011)	(−0.00050 to 0.00003)	.08

Abbreviations: CI, confidence interval; HIV, human immunodeficiency virus; hsCRP, high-sensitivity C-reactive protein; ICAM, intercellular adhesion molecule; IP-10, interferon γ-inducible protein; sCD14, soluble CD14; SE, standard error; TDF, tenofovir disoproxil fumarate; TNF, tumor necrosis factor; VCAM, vascular cell adhesion molecule. Bolded values represent significant P values, P ≤ .05.

^a^Linear mixed-effects model was used to examine the effect of the baseline albumin and statin on selected biomarkers. The top line regresses the described biomarker over 96 weeks on baseline serum albumin (g/dL), the shaded line regresses the same biomarker over 96 weeks and baseline serum albumin, controlling for statin use (group of randomization) over time. Slopes were examined to test group by time interactions.

^b^Statistical collinearity encountered when assessing time interaction variable and statin use for hsCRP, convergence could not be achieved.

In a separate multivariable model, after adjusting for CVD risk factors and factors known to affect serum albumin levels such as age, gender, smoking, BMI, creatinine clearance, and LDL cholesterol, every 1 g/dL decrease in baseline serum albumin remained associated with a 0.05-mm increase in cIMT over 96 weeks (*P* = .05; 95% confidence interval, −0.1071 to −0.0004 mm). In this model, lower serum albumin was associated with older age. Interleukin-6, hsCRP, and statin therapy were added to separate analyses that did not attenuate the association; however, the association between baseline serum albumin and cIMT over 96 weeks was no longer statistically significant. When stratifying baseline serum albumin by quartiles, the lowest level of baseline serum albumin remained associated with highest level of cIMT after adjusting for age, sex, IL-6, and statin therapy (Table 3).

**Table 3.** Multivariable Adjusted Analysis for Risk of Increased in Carotid Intima Media Thickness

Outcome	Parameter	β^ (SE)	95% CI of β^	*P* Value
Model 1^a^				
cIMT over 96 weeks (mm)	Baseline serum albumin (g/dL)	−0.05 (0.03)	(−0.106 to −0.0004)	**.05**
	Age (year)	0.007 (0.001)	(0.005 to 0.009)	**<.001**
	Sex (male vs female)	−0.04 (0.02)	(−0.08 to 0.01)	.14
	Current smoking	−0.009 (0.012)	(−0.03 to 0.01)	.45
	Body mass index (kg/m^2^)	−0.0006 (0.0013)	(−0.0032 to 0.0020)	.65
	Creatinine clearance (mL/min)	0.0001 (0.0002)	(−0.0002 to 0.0005)	.53
	LDL cholesterol (mg/dL)	0.00009 (0.00012)	(−0.00015 to 0.00033)	.45
Model 2^b^				
cIMT over 96 weeks (mm)	Baseline serum albumin (g/dL)	−0.05 (0.03)	(−0.110 to 0.003)	.06
	Age (year)	0.0070 (0.0009)	(0.0052–0.0088)	**<.001**
	Sex (male vs female)	−0.04 (0.02)	(−0.081 to 0.009)	.12
	hsCRP (μg/mL)	−0.0006 (0.0007)	(−0.002 to 0.0008)	.42
	Statin	−0.0002 (0.0001)	(−0.0005 to 0.00001)	.06
Model 3^c^				
cIMT over 96 weeks (mm)	Baseline serum albumin (g/dL)	−0.05 (0.03)	(−0.102 to 0.008)	.09
	Age (year)	0.007 (0.0009)	(0.005 to 0.009)	**<.001**
	Sex (male vs female)	−0.04 (0.02)	(−0.081 to 0.009)	.12
	IL-6 (pg/mL)	0.0005 (0.0004)	(−0.0002 to 0.0012)	.19
	Statin	−0.0002 (0.0001)	(−0.00051 to 0.00002)	.07
Categorizing Baseline Albumin by 4 Quartilesd				
cIMT over 96 weeks (mm)	Albumin (low vs lower middle)	−0.05 (0.02)	(−0.11 to −0.01)	**.02**
	Albumin (low vs higher middle)	−0.07 (0.03)	(−0.13 to −0.01)	**.02**
	Albumin (low vs high)	−0.05 (0.02)	(−0.096 to 0.001)	.06
	Age (year)	0.0068 (0.0009)	(0.005–0.009)	**<.001**
	Sex (male vs female)	−0.04 (0.02)	(−0.083 to 0.005)	.08
	IL-6 (pg/mL)	0.0005 (0.0004)	(−0.0002 to 0.0011)	.20
	Statin	−0.0003 (0.0001)	(−0.00051 to 0.00002)	.07

Abbreviations: BMI, body mass index; CI, confidence interval; cIMT, carotid intima media thickness; hsCRP, high-sensitivity C-reactive protein; IL, interleukin; LDL, low-density lipoprotein.

^a^Model 1 regresses baseline serum albumin on IMT over 96 weeks, controlling for age, sex, current smoking, BMI, creatinine clearance, and LDL. Bolded values represent significant P values, P ≤ .05.

^b^Model 2 regresses baseline serum albumin on IMT over 96 weeks, controlling for age, sex, hsCRP, and statin use (randomization group) over the study period.

^c^Model 3 regresses baseline serum albumin on IMT over 96 weeks, controlling for age, sex, IL-6 and statin use (randomization group) over the study period.

^d^Model 4 regresses baseline serum albumin, as categorized by quartiles (<3.8 g/dL [low]; 3.8–4.1 g/dL [lower middle]; 4.1 g/d–4.3 g/dL [higher middle]; and >4.3 g/dL [high]), on IMT over 96 weeks, controlling for age, sex, IL-6, and statin use (randomization group) over the study period.

## DISCUSSION

This is the first study to comprehensively assess the mechanisms by which serum albumin predicts clinical outcomes in treated HIV. We found that lower serum albumin is a predictor of increased atherosclerotic vascular risk over time, independent of traditional risk factors and statin therapy. We found that serum albumin is also associated with markers of systemic inflammation and hypercoagulation despite controlling for randomization to statin therapy, but not microbial translocation or gut integrity markers.

### Albumin and Atherosclerosis

In the HIV-uninfected population, epidemiological evidence indicate that hypoalbuminemia is associated with coronary artery disease, heart failure, atrial fibrillation, and stroke [13]. Malnutrition and inflammation are thought to contribute to the occurrence of hypoalbuminemia in these settings [21, 22]. However, CVD seems to remain inversely related to serum albumin even after adjusting for BMI and inflammation [1, 2].

In HIV, recent data revealed similar findings: low serum albumin seem to predict the risk of future morbidity. In the START trial, individuals with CD4 cell count >500 cells/µL were randomized to immediate versus delayed ART initiation. In these 4576 healthy adults with early stage HIV infection, serum albumin predicted future non-AIDS-related events (mainly non-AIDS cancer and CVD events) and hospitalizations [17]. This association remained after adjusting for traditional CVD risk factors, HIV-specific risk factors, and physiologic factors that affect serum concentrations of albumin (liver and renal functions). Our data support these findings. Low serum albumin was associated with a significant increase of cIMT, a measure of subclinical atherosclerosis that predicts future CVD outcomes in the general population [23]. We found that every 1 g/dL decrease in baseline serum albumin was associated with a 0.05-mm increase in cIMT over 2 years. This is a clinically meaningful increase considering that in uninfected adults, each 0.03-mm increase per year in cIMT has been associated with a 2-fold increase in coronary death and 3-fold increase in coronary events [23]. The association between serum albumin albumin and cIMT remained even after adjusting for traditional CVD risk factors including smoking. The D:A:D study [18] and 2 other epidemiological studies conducted in the general population [24, 25] have shown an interaction between smoking and serum albumin. In these studies, it appears that serum albumin primarily predicted outcome in individuals exposed to tobacco smoke. We controlled for smoking in our multivariable linear mixed-effects models, and the association between serum albumin and cIMT remained. In addition, there was no association between serum albumin and current smoking. We did not stratify further by smoking status because this would reduce sample size. Similarly in another earlier study in an uninfected elderly cohort, serum albumin was only associated with CVD among individuals with high levels of cholesterol. In our study, we did not find an association between serum albumin, LDL cholesterol, and cIMT.

We have previously shown that rosuvastatin substantially slows the progression of cIMT in this cohort over 96 weeks [20]. The association between baseline serum albumin and cIMT over 96 weeks remained significant after adjusting for statin therapy. The lowest levels of baseline serum albumin remained statistically significant predictors of elevated cIMT after adjusting for statin therapy in addition to the marker of systemic inflammation IL-6. Other studies have also found that serum albumin has remained associated with serious non-AIDS events and CVD mortality after adjusting for markers of systemic inflammation both in the HIV-infected population [17] and in uninfected older men [26], respectively. These imply that low serum albumin may be capturing more than a chronic inflammatory state.

### Serum Albumin’s Properties and Inflammation

Serum albumin has many physiologic properties including acting as a major circulating antioxidant [27]. Serum albumin is able to bind to many ligands including copper and iron, long-chain fatty acids, bilirubin, and homocysteine, preventing them from contributing to oxidized reactions, which are known risk factors for atherosclerosis [9]. The second mechanism is related to the antioxidant effect of serum albumin and its ability to help scavenge reactive oxygen species [13]. Serum albumin also has the capacity to bind lipopolysaccharides, other bacterial proteins, and other mediators of inflammation [28]. The relationship between serum albumin and inflammation is complex, and it appears that serum albumin could play a role in either stimulating or moderating immune activation depending on pathophysiological condition [29, 30]. As such, we attempted to determine whether low serum albumin could be representing other hallmarks of HIV associated with comorbidities and aging such as monocyte activation, T-cell activation, and microbial translocation or disruption in gut integrity.

Our findings indicate that low serum albumin is associated with inflammation and hypercoagulation over time in treated HIV infection. This relationship does not appear to be a consequence of disruption in gut integrity and microbial translocation because we did not find any statistically significant associations between serum albumin and zonulin-1 (a marker of gut epithelial cell function), I-FABP (a marker of enterocyte death), and LBP (a marker of microbial translocation). We have previously shown that statin reduces markers of inflammation, specifically T-cell and monocyte activation [19]. The associations between biomarkers and baseline serum albumin remained even after adjusting for statin use.

The relationship between serum albumin and inflammation may be bidirectional. Low serum albumin may be a driver or a consequence of ongoing inflammation in HIV. Our findings suggests that serum albumin is a marker of persistent inflammation in HIV, or it might be on the same causal pathway of inflammation and atherogenesis in HIV but further downstream.

Only a small fraction of our participants (3%) had hypoalbuminemia (serum albumin <3.5 g/dL), similar to the START study (2%) and the D:A:D trial (6%), suggesting that these patients are relatively healthy overall and would unlikely benefit from nutritional support to increase serum albumin.

### Strengths and Limitations

Major strengths of our study include the extensive immunophenotyping of our participants and longitudinal design. Our study has several limitations. First, the study was not initially designed to assess changes in serum albumin and may be underpowered to detect such changes. Moreover, the follow up may not have been long enough to address this due to the long half-life of serum albumin and the fact that changes in serum albumin under the study condition may occur very slowly (in contrast to sepsis and other acute conditions). We also did not measure hard clinical outcomes, and we used cIMT as a surrogate marker of atherosclerosis. Finally, our study included mostly men and African Americans; therefore, the findings may not be generalizable to other populations.

## CONCLUSIONS

To our knowledge, this is the first study to extensively investigate the inflammatory pathways associated with low serum albumin in treated HIV infection. Our results show that low serum albumin is associated with higher markers of chronic inflammation and hypercoagulation. Low serum albumin, in virally suppressed participants, also predict progression of subclinical atherosclerosis over time.

Further studies are warranted to determine whether albumin could be used as an easy marker to stratify patients who would benefit from newer anti-inflammatory statins to mitigate CVD in treated HIV. This is clinically relevant because serum albumin is routinely obtained in most HIV clinical centers as part of the clinical chemistry profile.

## Notes

Presented in part: ID Week, October 2018, San Francisco, CA.
